# Proposal of Allocatenococcus gen. nov., Allocoenonia gen. nov. and Allofournierella gen. nov. as replacement names for the illegitimate prokaryotic generic names Catenococcus Sorokin 1994, Coenonia Vandamme et al. 1999 and Fournierella Togo et al. 2017, respectively

**DOI:** 10.1099/ijsem.0.006547

**Published:** 2024-10-10

**Authors:** Aharon Oren, Eduardo A. Molinari Novoa

**Affiliations:** 1 Department of Plant and Environmental Sciences, The Institute of Life Sciences, The Hebrew University of Jerusalem, Edmond J. Safra Campus, Jerusalem 9190401, Israel; 2 Chess Consulting & Project, Lima 15039, Peru; 3 ‘La Molina’ National Agrarian University, Lima 15024, Peru

**Keywords:** *Catenococcus*, *Coenonia*, *Fournierella*, illegitimate names

## Abstract

The prokaryotic generic names *Catenococcus* Sorokin 1994, *Coenonia* Vandamme *et al*. 1999 and *Fournierella* Togo *et al*. 2017 are illegitimate because they are later homonyms of *Catenococcus* Hindák 1977, *Coenonia* Van Tieghem 1884 and *Fournierella* Collignon 1966, respectively (Principle 2 and Rules 51b(5) and 51b(4) of the International Code of Nomenclature of Prokaryotes). We, therefore, propose the replacement generic names *Allocatenococcus*, *Allocoenonia* and *Allofournierella*, with type species *Allocatenococcus thiocycli*, *Allocoenonia anatina* and *Allofournierella massiliensis*, respectively.

According to Rule 51b(4) of the International Code of Nomenclature of Prokaryotes (ICNP), a name or combination validly published before 31 December 2000 is illegitimate when it is a later homonym of a name of a taxon of prokaryotes, fungi, algae, protozoa or viruses. According to Principle 2 and Rule 51b(5) of the ICNP, a name or combination validly published on or after 1 January 2001 is illegitimate when it is a later homonym of a name or combination validly published or available under the International Code of Nomenclature for algae, fungi, and plants or the International Code of Zoological Nomenclature [1]. Based on these rules, we identified three illegitimate prokaryote genus names, *Catenococcus* Sorokin 1994, *Coenonia* Vandamme *et al*. 1999 and *Fournierella* Togo *et al*. 2017. We here propose replacement names for these genera.

## Illegitimacy of the name *Catenococcus* Sorokin 1994

The prokaryotic genus *Catenococcus* Sorokin 1994 (*Vibrionaceae*, ‘*Vibrionales*’, *Gammaproteobacteria*, *Pseudomonadota*) was described by Sorokin in 1992 [2,3]. The type species and thus far only species is *Catenococcus thiocycli* corrig. Sorokin 1994.

The generic name *Catenococcus* is a later homonym of *Catenococcus* Hindák 1977, the name of a genus of chlorococcalean algae, with a single species *Catenococcus tortuosus* [4]. Based on Rule 51b(4) of the ICNP [1], the name *Catenococcus* proposed for a prokaryotic genus is therefore illegitimate. In accordance with Rule 54, we here propose a replacement name.

## Description of *Allocatenococcus* Oren and Molinari Novoa gen. nov.

*Allocatenococcus* (Al.lo.ca.te.no.coc’cus. Gr. masc. adj. *allos*, other; N.L. masc. n. *Catenococcus*, a genus name of chlorococcalean algae; N.L. masc. n. *Allocatenococcus*, another *Catenococcus*).

Earlier illegitimate homotypic synonym: *Catenococcus* Sorokin 1994.

The description of the genus is as given for *Catenococcus* [2].

The type species is *Allocatenococcus thiocycli* (Sorokin 1994) Oren and Molinari Novoa.

## Description of *Allocatenococcus thiocycli* (Sorokin 1994) Oren and Molinari Novoa comb. nov.

*Allocatenococcus thiocycli* (thi.o.cyc’li. Gr. neut. n. *theion*, sulfur (Latin transliteration *thium*); L. masc. n. *cyclus*, circle, cycle; N.L. gen. n. *thiocycli*, of a sulfur circle).

Illegitimate basonym: *Catenococcus thiocycli* corrig. Sorokin 1994.

The description of the species is as given for *Catenococcus thiocycli* corrig. [2].

The type strain is TG 5-3^T^ (= ATCC51228ATCC 51228^T^=DSM 9165DSM9165^T^=NCCB 92012), isolated from a near-shore sulfidic hydrothermal area in Matupy Harbour of New Britain Island, Papua New Guinea.

The GenBank/EMBL/DDBJ accession number for the 16S rRNA gene sequence of the type strain is AF139723.

## Illegitimacy of the name *Coenonia* Vandamme *et al*. 1999

The prokaryotic genus *Coenonia* Vandamme *et al*. 1999 (*Flavobacteriaceae*, *Flavobacteriia*, *Bacteroidota*) was described by Vandamme *et al*. in 1999 [5]. The type species and thus far only species is *Coenonia anatina* Vandamme *et al*. 1999.

The generic name *Coenonia* is a later homonym of *Coenonia* Van Tieghem 1884, the name of a genus of protozoa (Mycetozoa, Dictyosteliomycetes) [6]. Based on Rule 51b(4) of the ICNP [1], the name *Coenonia* proposed for a prokaryotic genus is therefore illegitimate. In accordance with Rule 54, we here propose a replacement name.

## Description of *Allocoenonia* Oren and Molinari Novoa gen. nov.

*Allocoenonia* (Al.lo.coe.no’ni.a. Gr. masc. adj. *allos*, other; N.L. fem. n. *Coenonia*, a genus name of protozoa; N.L. fem. n. *Allocoenonia*, another *Coenonia*).

Earlier illegitimate homotypic synonym: *Coenonia* Vandamme *et al*. 1999.

The description of the genus is as given for *Coenonia* [5].

The type species is *Allocoenonia anatina* (Vandamme *et al*. 1999) Oren and Molinari Novoa.

## Description of *Allocoenonia anatina* (Vandamme *et al.* 1999) Oren and Molinari Novoa comb. nov.

*Allocoenonia anatina* (a.na.ti’na. L. fem. adj. *anatina*, of or pertaining to the duck).

Illegitimate basonym: *Coenonia anatina* Vandamme *et al*. 1999.

The description of the species is as given for *Coenonia anatin*a [5]. *Coenonia anatina* Vandamme *et al*. 1999 was previously known as *Riemerella anatipestifer*-like taxon 1502 [7].

The type strain is 1502-91^T^ (=CCUG 46148CCUG46148^T^=CIP 106119CIP106119^T^=DSM 25327DSM25327^T^=LMG 14382LMG14382^T^), isolated from a Pekin duck in Germany.

The GenBank/EMBL/DDBJ accession number for the 16S rRNA gene sequence of the type strain is Y17612.

The basonym was assigned to Risk Group 2 [German TRBA 466, ‘Einstufung von Prokaryonten (Bacteria und Archaea) in Risikogruppen’].

## Illegitimacy of the name *Fournierella* Togo *et al*. 2017

The prokaryotic genus *Fournierella* Togo *et al*. 2017 (*Oscillospiraceae*, *Eubacteriales*, *Clostridia*, *Bacillota*) was described by Togo *et al*. in 2017 [8]. The type species and thus far only species is *Fournierella massiliensis* Togo *et al*. 2017. In addition, a number of *Candidatus* species have been assigned to the genus: ‘Candidatus Fournierella excrementigallinarum’‘Candidatus Fournierella merdavium’, Candidatus Fournierella merdigallinarum’‘Candidatus Fournierella merdipullorum’‘*Candidatus* Fournierella excrementigallinarum’, ‘*Candidatus* Fournierella merdavium’, ‘*Candidatus* Fournierella merdigallinarum’, ‘*Candidatus* Fournierella merdipullorum’, ‘*Candidatus* Fournierella pullicola’ and *‘Candidatus* Fournierella pullistercoris’ [9,10].

The generic name *Fournierella* Togo *et al*. 2017 is a later homonym of the name *Fournierella* Collignon 1966, a fossil ammonite (Mollusca, Cephalopoda), described originally as a subgenus of *Pseudoschloenbachia* Spath [11,12], later treated as of generic status [13]. According to the International Code of Zoological Nomenclature Principle of Coordination (Art. 43.1), ‘A name established for a taxon at either rank in the genus group is deemed to have been simultaneously established by the same author for a nominal taxon at the other rank in the group’ [14]. Thus, both *Pseudoschloenbachia* subgen. *Fournierella* and *Fournierella* have the same author, date and place of publication.

Since *Fournierella* Collignon is available within the zoological nomenclature as a genus, the name *Fournierella* proposed for a prokaryotic genus is illegitimate based on Principle 2 and Rule 51b(5) of the ICNP. In accordance with Rule 54, we here propose a replacement name.

## Description of *Allofournierella* Oren and Molinari Novoa gen. nov.

*Allofournierella* (Al.lo.four.nier.el’la. Gr. masc. adj. *allos*, other; N.L. fem. n. *Fournierella*, a genus name of fossil ammonites; N.L. fem. n. *Allofournierella*, another *Fournierella*).

Earlier illegitimate homotypic synonym: *Fournierella* Togo *et al*. 2017.

The description of the genus is as given for *Fournierella* [8].

The type species is *Allofournierella massiliensis* (Togo *et al*. 2017) Oren and Molinari Novoa.

## Description of *Allofournierella massiliensis* (Togo *et al.* 2017) Oren and Molinari Novoa comb. nov.

*Allofournierella massiliensis* (mas.si.li.en’sis. L. fem. adj. *massiliensis*, of Massilia, the Latin name of Marseille).

Basonym: *Fournierella massiliensis* Togo *et al*. 2017.

The description of the species is as given for *Fournierella massiliensis* [8].

The type strain is AT2^T^ (=CSUR P2014CSURP2014^T^=DSM 100451DSM100451^T^), isolated from the faeces of a healthy 28-year-old French male.

The GenBank/EMBL/DDBJ accession numbers for the genome sequence and 16S rRNA gene sequence of strain AT2^T^ are FAUK00000000 and LN846908, respectively.

The *Candidatus* Fournierella species described by Gilroy *et al.* [9,10] should therefore be renamed ‘Candidatus Allofournierella excrementigallinarum’‘Candidatus Allofournierella merdavium’, ‘Candidatus Allofournierella merdigallinarum’‘Candidatus Allofournierella merdipullorum’‘*Candidatus* Allofournierella excrementigallinarum’, ‘*Candidatus* Allofournierella merdavium’, ‘*Candidatus* Allofournierella merdigallinarum’, ‘*Candidatus* Allofournierella merdipullorum’, ‘*Candidatus* Allofournierella pullicola’ and *‘Candidatus* Allofournierella pullistercoris’. Their descriptions are as follows:

‘*Candidatus* Allofournierella excrementavium’ (ex.cre.ment.a’vi.um. L. neut. n. *excrementum*, excrement; L. fem. n. *avis*, bird; N.L. gen. n. *excrementavium*, of bird excrement).

The description is as given for ‘*Candidatus* Fournierella excrementavium’ by Gilroy *et al.* [9].

The type genome, MAG ID ChiHcec27-1717, is available via NCBI BioSample SAMN15816881.

‘*Candidatus* Allofournierella excrementigallinarum’ (ex.cre.men.ti.gal.li.na’rum. L. neut. n. *excrementum*, excrement; L. fem. n. *gallina*, hen; N.L. gen. n. *excrementigallinarum*, of hen excrement).

The description is as given for ‘*Candidatus* Fournierella excrementigallinarum’ by Gilroy *et al.* [9].

The type genome, MAG ID 1136, is available via NCBI BioSample SAMN15816650.

‘*Candidatus* Allofournierella merdavium’ (merd.a’vi.um. L. fem. n. *merda*, faeces; L. fem. n. *avis*, bird; N.L. gen. n. *merdavium*, of bird faeces).

The description is as given for ‘*Candidatus* Fournierella merdavium’ by Gilroy *et al.* [9].

The type genome, MAG ID ChiBcec4-1730, is available via NCBI BioSample SAMN15816653.

‘*Candidatus* Allofournierella merdigallinarum’ (mer.di.gal.li.na’rum. L. fem. n. *merda*, faeces; L. fem. n. *gallina*, hen; N.L. gen. n. *merdigallinarum*, of hen faeces).

The description is as given for ‘*Candidatus* Fournierella merdigallinarum’ by Gilroy *et al.* [9].

The type genome, MAG ID 6296, is available via NCBI BioSample SAMN15816675.

‘*Candidatus* Allofournierella merdipullorum’ (mer.di.pul.lo’rum. L. fem. n. *merda*, faeces; L. masc. n. *pullus*, a young chicken; N.L. gen. n. *merdipullorum*, of the faeces of young chickens).

The description is as given for ‘*Candidatus* Fournierella merdipullorum’ by Gilroy *et al.* [9].

The type genome, MAG ID ChiGjej4B4-18154, is available via NCBI BioSample SAMN15816693.

‘*Candidatus* Allofournierella pullicola’ (pul.li’co.la. L. masc. n. *pullus*, a young chicken; L. suff. –*cola*, inhabitant of; N.L. n. *pullicola*, an inhabitant of young chickens).

The description is as given for ‘*Candidatus* Fournierella pullicola’ by Gilroy *et al.* [9].

The type genome, MAG ID 2239, is available via NCBI BioSample SAMN15816745.

‘Candidatus Allofournierella pullistercoris’ (pul.li.ster’co.ris. L. masc. n. *pullus*, a young chicken; L. neut. n. *stercus*, dung; N.L. gen. n. *pullistercoris*, of young chicken faeces).

The description is as given for ‘*Candidatus* Fournierella pullistercoris’ by Gilroy *et al.* [9].

The type genome, MAG ID B5-2728, is available via NCBI BioSample SAMN15816762.

## Abbreviations

ICNP: International Code of Nomenclature of Prokaryotes
ICZN: International Code of Zoological Nomenclature
